# Effect of Substrates on Femtosecond Laser Pulse-Induced Reductive Sintering of Cobalt Oxide Nanoparticles

**DOI:** 10.3390/nano11123356

**Published:** 2021-12-10

**Authors:** Mizue Mizoshiri, Kyohei Yoshidomi, Namsrai Darkhanbaatar, Evgenia M. Khairullina, Ilya I. Tumkin

**Affiliations:** 1 Department of Mechanical Engineering, Nagaoka University of Technology, 1603-1, Kamitomioka, Nagaoka 9402142, Japan; s193097@stn.nagaokaut.ac.jp (K.Y.); s203050@stn.nagaokaut.ac.jp (N.D.); 2 Institute of Chemistry, Saint Petersburg State University, 7/9 Universitetskaya Nab., 199034 St. Petersburg, Russia; e.khayrullina@spbu.ru (E.M.K.); i.i.tumkin@spbu.ru (I.I.T.)

**Keywords:** femtosecond laser reductive sintering, printing, Co_3_O_4_ nanoparticle ink, cobalt/cobalt oxide composite, polymer substrate

## Abstract

Direct writing of cobalt/cobalt oxide composites has attracted attention for its potential use in catalysts and detectors in microsensors. In this study, cobalt-based composite patterns were selectively formed on glass, polyethylene naphthalate (PEN), and polyethylene terephthalate (PET) substrates via the femtosecond laser reductive sintering of Co_3_O_4_ nanoparticles in an ambient atmosphere. A Co_3_O_4_ nanoparticle ink, including the nanoparticles, ethylene glycol as a reductant, and polyvinylpyrrolidone as a dispersant, was spin-coated onto the substrates. Near-infrared femtosecond laser pulses were then focused and scanned across the ink films to form the patterns. The non-sintered nanoparticles were subsequently removed from the substrate. The resulting sintered patterns were found to be made up of Co/CoO composites on the glass substrates, utilizing various pulse energies and scanning speeds, and the Co/CoO/Co_3_O_4_ composites were fabricated on both the PEN and PET substrates. These results suggest that the polymer substrates with low thermal resistance react with the ink during the reductive sintering process and oxidize the patterns more easily compared with the patterns on the glass substrates. Such a direct writing technique of cobalt/cobalt oxide composites is useful for the spatially selective printing of catalysts and detectors in functional microsensors.

## 1. Introduction

There is considerable interest in the use of laser direct writing technologies in the fabrication of metal patterns for printed electronics because the techniques permit photolithography-free electrode fabrication. Recently, low-cost non-noble metal electrodes, such as Cu and Ni, have attracted attention as potential alternatives to Ag or Au electrodes. For example, it was shown that Cu and Ni microelectrodes can be sufficiently applied to the electrochemical detection of glucose [1]. Although many techniques, including inkjet, flexo, gravure, and screen printing, have been developed for the printed electronics process, the advantage of laser direct writing is that the patterning and the metallization can be performed simultaneously via the sintering or reductive sintering of nanoparticle inks. Cu nanoparticle inks have previously been coated and selectively sintered via irradiating continuous-wave and pulsed lasers on substrates [2,3,4]. Cu electroconductive patterns were formed on substrates after removing the non-sintered nanoparticles. An optimal laser scanning condition existed to obtain high electrical conductivity, which prevents the patterns from re-oxidizing or losses of energy [2]. However, the absorption of the laser light by the metal nanoparticle inks is generally low because of the high reflectance and scattering of the nanoparticles.

To increase the laser light absorption of the nanoparticles, metal oxide nanoparticles with small bandgaps have been used as the ink that makes up the metal oxide nanoparticles and reductant agents to increase the absorbance of the laser light [5,6,7,8]. In this method, the absorbed laser light induces reductive sintering of the metal oxide nanoparticles. In addition, the methods are generally vacuum-free processes of metallization. Cu patterns were formed via the reduction and sintering of copper oxide (I or II) nanoparticles mixed with a reductant. A CuO nanoparticle ink composed of CuO nanoparticles (<50 nm in diameter), ethylene glycol, and polyvinylpyrrolidone (PVP) was coated on glass and flexible polyimide (PI) substrates [5]. Then, near-infrared or green continuous-wave and nanosecond pulsed lasers were focused onto the CuO nanoparticle ink films to reduce and sinter the ink. Finally, the non-sintered nanoparticles were removed by rinsing the samples in deionized water. A Cu_2_O nanoparticle ink, including Cu_2_O nanoparticles, 2-propanol, and PVP, was also used for laser reductive sintering [6]. Formic acid was generated via the thermochemical reaction of 2-propanol and PVP.

Ni electrodes have also been fabricated via the laser reductive sintering of NiO nanoparticles [7,8]. The NiO nanoparticles mixed with toluene were reduced to Ni using protons supplied from the toluene molecules. To achieve this, the ink-deposited films were irradiated by green continuous-wave laser light in order to induce a thermochemical reduction in ambient conditions.

Femtosecond laser reductive sintering via single- and multi-photon absorption processes have also been developed to form Cu-based patterns [9,10,11,12,13]. In this process, thermal energy converted from the absorbed photon energy reduces the metal oxide nanoparticles. The femtosecond laser reductive sintering achieves non-equilibrium Cu-rich and Cu_2_O-rich patterns from copper oxide (I and II) nanoparticles by controlling the nature of the laser irradiation, including the laser scanning speed and pulse energy [9,10]. Additionally, high-purity Cu patterns without significant oxidation can be formed under inert gas injection [11]. CuO/NiO mixed nanoparticle inks have been selectively sintered to form p-type Cu_2_O/NiO-rich and n-type Cu-Ni-rich thermoelectric couples via careful control of the laser irradiation. It was found that p-type and n-type thermoelectric couples could be fabricated at low and high scanning speeds, respectively [12,13].

The ease with which metal oxide nanoparticles can be reductively sintered into a metal is determined by the standard free energy of formation of the metal oxide. Noble metal oxides, such as Ag and Au oxides, are easily reduced and sintered in air because the standard free energies of formation of the metal oxides are small (<200 kJ/mol). In contrast, Cu and Ni usually require an inert atmosphere for sintering. Therefore, when CuO and NiO are reduced to Cu and Ni, respectively, the use of reductant agents is necessary. However, other metal oxide nanoparticles have not been reported to be reduced to metals for patterning in air using the laser reductive sintering because the metal oxides are more stable than the metals.

Cobalt/cobalt oxide composites such as Co, CoO, and Co_3_O_4_, are promising materials for use in various applications and devices, including gas sensors, non-enzyme detectors, and energy storage [14,15,16]. We have already focused on the application of Co-based materials for the catalyst. Composites of bimetallic Cu-Co systems or other systems where the catalytic effect is enhanced by the presence of two metals have also been shown to be promising candidates for electrochemical microsensors [17,18]. If the direct writing of composites such as cobalt and cobalt oxides were achieved, the rapid printing device would likely play an important role in the Internet of Things. These kinds of the metal, metal oxides, and their composites are generally fabricated using well-established semiconductor technology and the conventional powder sintering process [15]. However, these processes need the vacuum atmosphere to control the oxidation. In the femtosecond laser direct writing process, those materials are expected to be easily generated in air because the femtosecond laser pulses easily generate non-equilibrium materials, such as various valences of metal oxides, because rapid heating and cooling are achieved in the reductive sintering process [19]. Therefore, femtosecond laser reductive sintering of Co_3_O_4_ has a high potential for selectively patterning Co and cobalt oxides by controlling the laser irradiation conditions.

In this study, we investigate the patterning properties of cobalt-based direct writing via the femtosecond laser reductive sintering of cobalt oxide nanoparticles. First, cobalt oxide nanoparticle inks are prepared and coated as films on various substrates, including glass and flexible polymer substrates, such as polyethylene naphthalate (PEN) and polyethylene terephthalate (PET), which are promising candidates for use as flexible devices [20]. Then, near-infrared femtosecond laser pulses are irradiated onto the metal oxide to generate metals. Cobalt-based patterns were fabricated via the femtosecond laser reductive sintering of Co_3_O_4_ nanoparticles.

## 2. Materials and Methods

### 2.1. Preparataion of the Co_3_O_4_ Nanoparticle Ink

A Co_3_O_4_ nanoparticle ink was prepared using commercially available Co_3_O_4_ nanoparticles (Sigma Aldrich, St. Louis, MO, USA, particles with a diameter <50 nm), ethylene glycol, and PVP (Mw ~10,000). The concentrations of the Co_3_O_4_ nanoparticles, ethylene glycol, and PVP were 33, 55, and 12 wt.%, respectively. The PVP was first mixed with ethylene glycol, and the Co_3_O_4_ nanoparticles were then mixed with PVP-ethylene glycol solution using ultrasonic waves.

### 2.2. Femtosecond Laser Direct Writing Process

**Figure 1:**
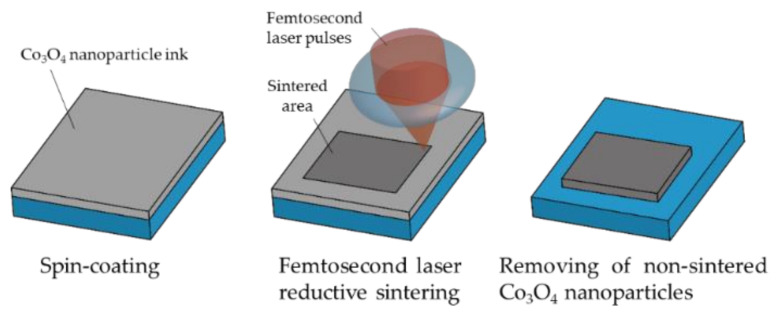
Procedure for the laser direct writing of cobalt/cobalt oxide composite materials using femtosecond laser reductive sintering of cobalt oxide nanoparticles.

The femtosecond laser direct writing process was made up of three steps: (i) the coating of the ink on the substrates, (ii) the femtosecond laser direct writing, and (iii) the removal of the non-sintered nanoparticles, and this process is shown schematically in Figure 1. In the coating step, the Co_3_O_4_ nanoparticle ink was spin-coated on various substrates, including glass, PEN, and PET. The thickness of the coated ink film was ~10 µm at the spinning rate of 7000 rpm, which allowed to form the patterns with adhesion on the substrates. The parameters of the glass, PEN, and PET substrates are shown in Table 1. By considering the thermal properties, such as thermal conductivity, heat capacity, and density of the substrates, the thickness of the PEN and PET substrates was determined to be approximately four times thinner than that of the glass substrates.

Femtosecond laser pulses with Gaussian distributions (M^2^ = 1.1) were then focused and scanned on the surface of the ink using an objective lens with a numerical aperture of 0.45. The focal spot on the ink surface was approximately 3 µm, measured using a knife edge method. The wavelength, pulse duration, and repetition frequency of the femtosecond laser pulses used were 780 nm, 120 fs, and 80 MHz, respectively. The laser pulse energy was varied across energies less than 0.74 nJ. The sample substrates were scanned to form arbitrary shape patterns using an XYZ-mechanical stage (ALIO INDUSTRIES, INC., Arvada, CO, USA). An air-flow system was used during the laser irradiation to avoid covering the surface of the objective lens with the vapors generated by the thermochemical reduction of the Co_3_O_4_. Considering the bandgap of Co_3_O_4_ (~1.5 eV) [24,25], the laser pulses with the near-infrared light are expected be absorbed by the cobalt oxide nanoparticle ink. Finally, following the irradiation procedure, any non-sintered nanoparticles on the substrates were removed by rinsing the substrate in ethylene glycol and ethanol. The ethylene glycol and PVP in the Co_3_O_4_ NP ink were expected to reduce Co_3_O_4_ to Co and CoO according to the following equations [5,15,26]:

2HO(CH_2_)_2_OH -> 2C_2_H_4_O + H_2_O(g) (1)

2C_2_H_4_O + Co_3_O_4_ -> 2C_2_H_4_O_2_ + 2H+ 2e^−^ + Co^2+^/Co^3+^ + O^2−^ -> Co/CoO + 2C_2_H_4_O_2_ + H_2_O(g). (2)

### 2.3. Evaluation of the Patterns and Substrates

The line width and the morphology of the resulting patterns were evaluated using an optical microscope (Keyence, Osaka, Japan, VHX-500F) and scanning electron microscopy (Hitachi, Tokyo, Japan, FlexSEM 1000 II). The crystal structures of the sintered patterns were examined using an X-ray powder diffractometer (Rigaku Corporation, Tokyo, Japan, MiniFlex) using Cu-Kα radiation. The composition of the fabricated patterns was also analyzed using an energy dispersive X-ray spectrometer (Oxford Instruments, Abingdon, UK) attached to the SEM. Cross-sectional profiles of both the line patterns and raster scanned patterns were measured using a stylus profiler (Ulvac Inc., Chigasaki, Japan, Dektak 6M). The damages of the substrates were examined using attenuated total reflection Fourier transform infrared spectroscopy (ATR-FTIR spectroscopy, Shimadzu Corp., Kyoto, Japan, Affinity-1).

## 3. Results and Discussion

### 3.1. Absorption Properties of the Co_3_O_4_ Nanoparticle Ink

**Figure 2:**
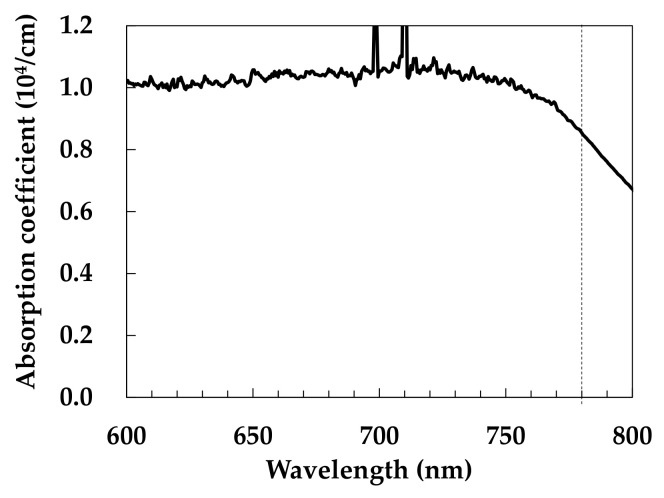
Absorption spectrum of the Co_3_O_4_ nanoparticle ink film coated on the SiO_2_ glass substrate.

Figure 2 shows the absorption properties of the Co_3_O_4_ nanoparticle ink film coated on a SiO_2_ glass substrate. The intense absorption at the wavelength of 780 nm indicates that the femtosecond laser pulses of the near-infrared light were absorbed via a single-photon absorption process.

### 3.2. Line Width

**Figure 3:**
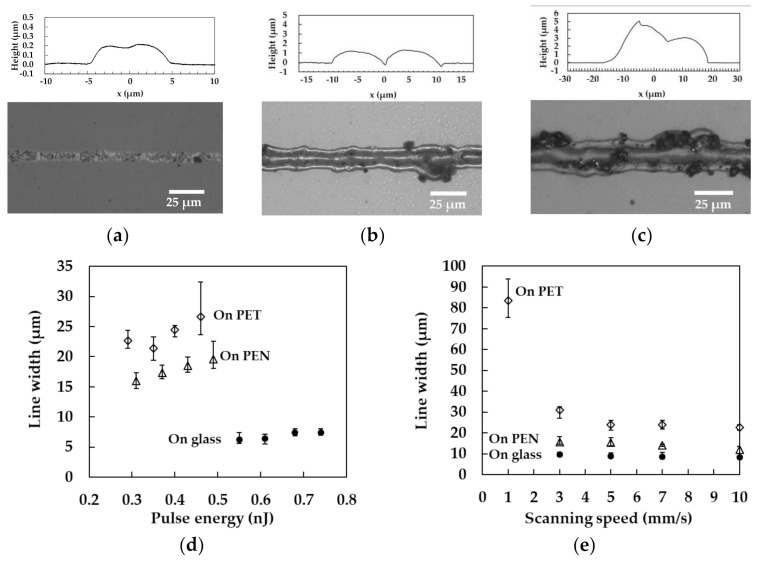
(**a**–**c**) Optical microscope images and cross-sectional profiles of the minimum line patterns fabricated on glass, PEN, and PET substrates, respectively. Dependences of the line width of the patterns on (**d**) the pulse energy at a scanning speed of 5 mm/s and (**e**) the scanning speed. The data points shown correspond to energies of 0.29, 0.31, and 0.68 nJ on PET, PEN, and glass substrates, respectively.

Line patterns were formed on various substrates with the aim of evaluating the effect of the substrates on the line width. Figure 3a–c show optical microscope images and the cross-sectional profiles of the minimum line patterns fabricated on glass, PEN, and PET substrates. Continuous line patterns were formed on all the substrates. The minimum pulse energy on PEN substrates required for the continuous line patterning was higher than that on PET substrates. The heat capacity of PEN is higher than that of PET, even though the other parameters such as thermal conductivity, density, and melting point are also the same. As a result, higher pulse energy was thought to be required for the patterning on PEN than on PET. These results were consistent with the Cu precipitation using femtosecond laser pulses on glass and polydimethylsiloxane substrates [27]. The heights of the center of the lines was lower than both sides, suggesting that the high peak intensity of the laser pulses decreased the height because of the high dense sintering. The properties are thought to be caused by femtosecond laser pulses with the Gaussian profile, which are the same shapes reported previously [28]. The finer patterns were formed on glass substrates even though the pulse energy was higher than that on PEN and PET. These results indicate that the low thermal conductivity of PEN and PET achieved a higher temperature than that on glass substrates. All the line patterns were wider than the focal spot diameter, indicating that the thermal diffusion increased the line width.

### 3.3. Crystal Structures of the Patterns on Various Substrates

**Figure 4:**
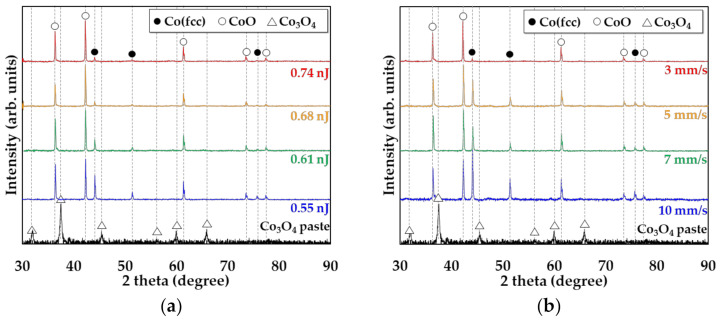
Crystal structures of the patterns fabricated on glass substrates (**a**) at a scanning speed of 5 mm/s and a pulse energy of 0.55–0.74 nJ, and (**b**) a scanning speed in the range 3–10 mm/s with a pulse energy of 0.55 nJ.

The crystal structures of the patterns fabricated on the glass substrates are shown in Figure 4. The patterns of 5 × 3 mm^2^ were fabricated by raster scanning of the focal spot. The raster pitch was determined to be 5 µm by considering the line width shown in Figure 3. The dependence of the crystal structures on the pulse energy for a scanning speed of 5 mm/s is shown in Figure 4a. The patterns fabricated with a pulse energy of more than 0.55 nJ adhered to the glass substrates after rinsing the samples, which was undertaken to remove the non-sintered nanoparticles. The X-ray diffraction (XRD) peaks corresponding to the fcc-Co increased with decreasing pulse energy. In contrast, the XRD peaks corresponding to CoO increased with increasing pulse energy. The peaks corresponding to Co_3_O_4_ disappeared after the laser irradiation. The dependence of the crystal structures on the scanning speed is shown in Figure 4b. The fcc-Co generation increased with increasing scanning speed, whereas the generation of CoO increased with decreasing scanning speed. These results suggest that increasing the irradiation energy increases the generation of CoO. The Co_3_O_4_ nanoparticles were reduced via the following process [26]:

Co_3_O_4_ -> Co(fcc) (reduced) + CoO (re-oxidized) (in air) (3)

**Figure 5:**
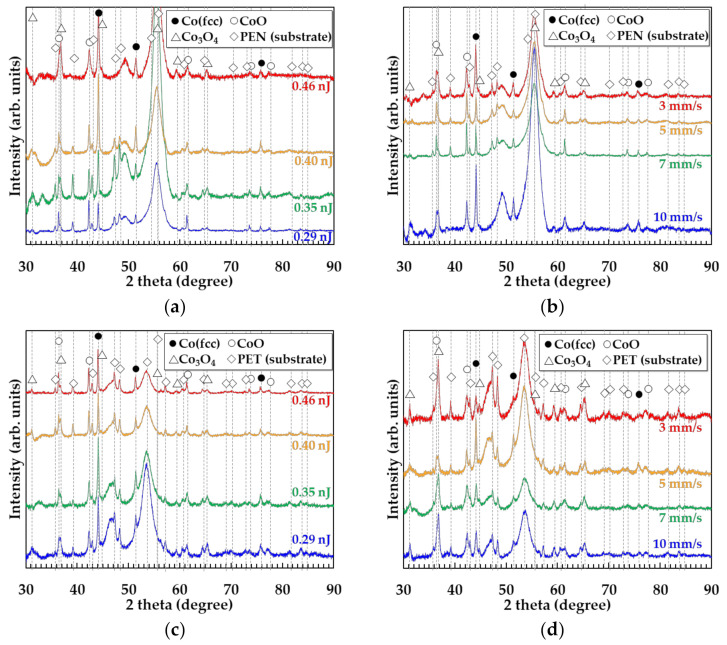
Crystal structures of the patterns fabricated on the PEN substrates (**a**) at a scanning speed of 5 mm/s and pulse energy in the range 0.29–0.46 nJ, and (**b**) with a scanning speed in the range 3–10 mm/s and a pulse energy of 0.29 nJ. Crystal structures of the patterns fabricated on PET substrates (**c**) at a scanning speed of 5 mm/s and pulse energy in the range 0.29–0.46 nJ, and (**d**) with a scanning speed in the range 3–10 mm/s and a pulse energy of 0.29 nJ.

**Figure 6:**
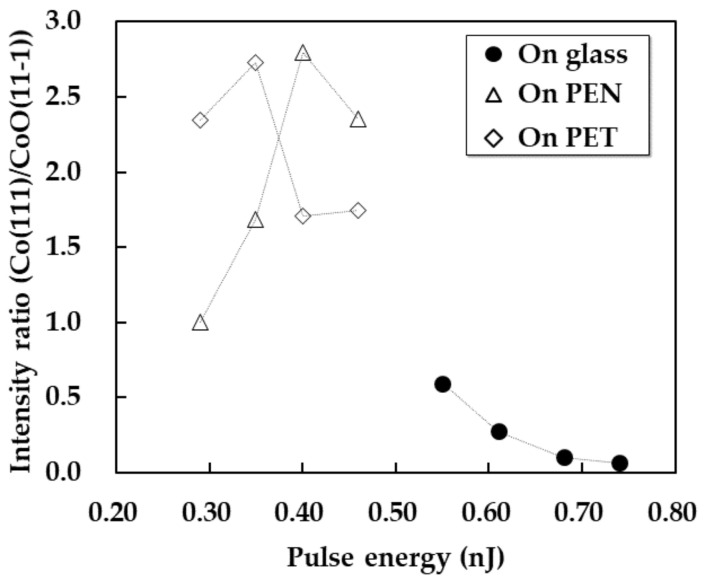
Intensity ratio of Co(111)/CoO(111) of the patterns fabricated at a scanning speed of 5 mm/s on the glass, PEN, and PET substrates.

To evaluate the effect of the substrate material on the crystal structures of the patterns, the crystal structures of the patterns on the PEN and PET substrates were investigated, as shown in Figure 5. The patterns of 5 × 3 mm^2^ were fabricated by raster scanning with the raster pitch of 5 µm. The patterns formed on the PET and PEN substrates remained on the substrate after rinsing even at low pulse energy, unlike those on the glass substrates. These results suggest that PEN and PET substrates with low thermal resistance and low thermal conductivity improved the adhesion of the patterns to the substrate by achieving high temperatures and reacting with the patterns. In addition, Co_3_O_4_ was observed in the patterns on the PEN and PET substrates, whereas it was not observed in the patterns on the glass substrates. To compare the oxides in the patterns on the glass, PEN, and PET substrates, the generation ratios of Co and CoO in the patterns are shown in Figure 6. The Co content in the patterns on the glass substrates increased by preventing the reoxidation by using a low pulse energy; in the case of the PET and PEN substrates, the intensity ratios of Co/CoO exhibited peaks at a pulse energy of 0.43 and 0.31 nJ, respectively. These results suggest that the PET and PEN substrates with low thermal resistance and containing oxygen reacted with the patterns and easily generated oxides, including Co_3_O_4_ and CoO. Furthermore, the balance between the reduction and reoxidation caused the different composition ratios of Co/CoO depending on the pulse energy, which has been reported previously in femtosecond laser reductive sintering of CuO nanoparticles [19]. The reason why the Co was generated at a lower pulse energy on PET than on PEN was thought to be that PET was heated easier than PEN because its heat capacity is smaller than that of PEN, even though the other parameters are almost the same, as shown in Table 1. In addition, the pulse energy for pattering on PEN and PET substrates was lower than that on glass substrates to prevent melting the polymer substrates. As a result, CoO in the patterns on PET and PEN substrates possibly came from the reduction of Co_3_O_4_ and reoxidation of Co, and Co_3_O_4_ possibly came from raw materials and reoxidation of the reduced Co_3_O_4_. The cobalt/cobalt oxide composites were expected to be generated in an oxygen-rich atmosphere:

Co_3_O_4_ -> Co(fcc) (reduced) + CoO (reduced/re-oxidized) + Co_3_O_4_ (remained/re-oxidized) (in air).(4)

### 3.4. Damage of the Substrates

**Figure 7:**
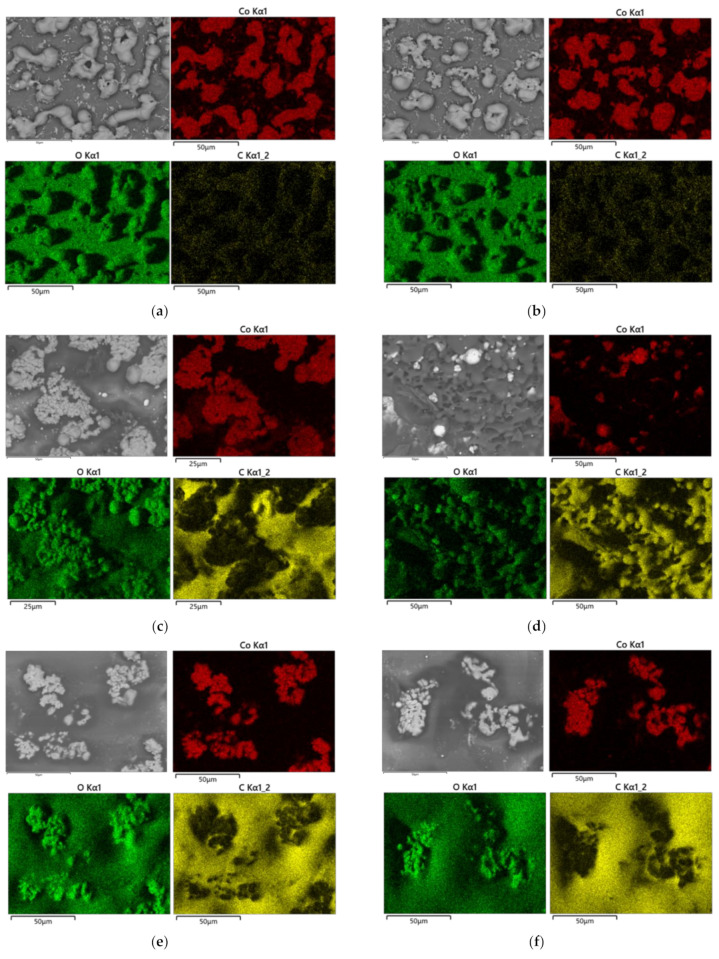

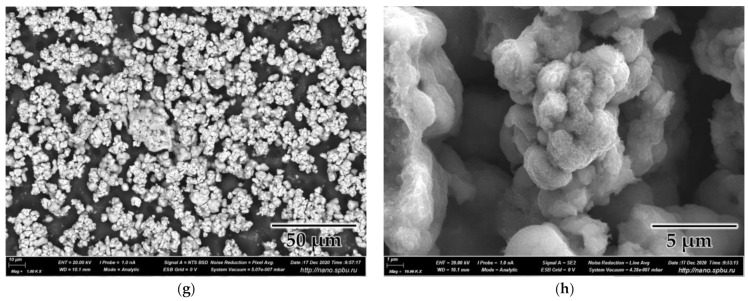
SEM images and EDS mappings of the patterns fabricated on glass, PEN, and PET substrates using a scanning speed of 5 mm/s, and a laser pulse energy of (**a**) 0.55 nJ and (**b**) 0.74 nJ on glass substrates, (**c**) 0.29 nJ and (**d**) 0.46 nJ on PEN substrates, and (**e**) 0.29 nJ and (**f**) 0.46 nJ on PET substrates. (**g**) FE-SEM image of the patterns fabricated on PEN substrates using a scanning speed of 5 mm/s and a laser pulse energy of 0.29 nJ, and (**h**) an enlarged image of (**g**).

Figure 7 shows SEM images and EDS mappings of the patterns (5 × 3 mm^2^) fabricated on the glass, PEN, and PET substrates, which were the same samples shown in Section 3.3. The scanning speed was 5 mm/s. The pulse energy was 0.55 and 0.74 nJ on the glass substrates, and 0.29 and 0.46 nJ on PEN and PET substrates, which generated minimum and maximum line width at a scanning speed of 5 mm/s, respectively. Both cobalt and oxygen elements were observed in the same area of the glass substrates, although carbon was not observed in significant quantities (Figure 7a,b). These results indicate that the PVP in the ink was vapored during the reductive sintering process. Observing the crystal structure shown in Figure 4, we conclude that the Co/CoO composite patterns were formed via the reductive sintering of Co_3_O_4_ nanoparticles. In contrast, the PEN and PET substrates were seen to be significantly damaged, which was different from the glass substrates. To confirm that the patterns were fabricated by sintering under specific conditions, FE-SEM images are shown in Figure 7g,h. The patterns were fabricated on the PEN substrate using a scanning condition of 5 mm/s and a pulse energy of 0.29 nJ, which was the same as the patterns shown in Figure 7c. Unlike the patterns on glass substrates, the Co_3_O_4_ nanoparticles were sintered, not melted, on PEN substrates. The patterns exhibited electrical conductivity. The resistance of the pattern (5 × 3 mm^2^) was approximately 500 Ω (length: 5 mm) due to the presence of the oxide phase, as well as due to the high porosity. It should be noted that such values are quite suitable for the use of the synthesized structures as microelectrodes for electrochemical analysis of bioanalysis. The resistance measured a couple of months later was almost the same as that of as-prepared structures, indicating that the outermost surface of the precipitation was stable cobalt oxides. The adhesion of the patterns was also evaluated simply using the taping test. The patterns remained on PEN and PET substrates, although the patterns on glass substrates did not survive. In addition, the patterns on PEN and PET substrates survived after bending a few times.

**Figure 8:**
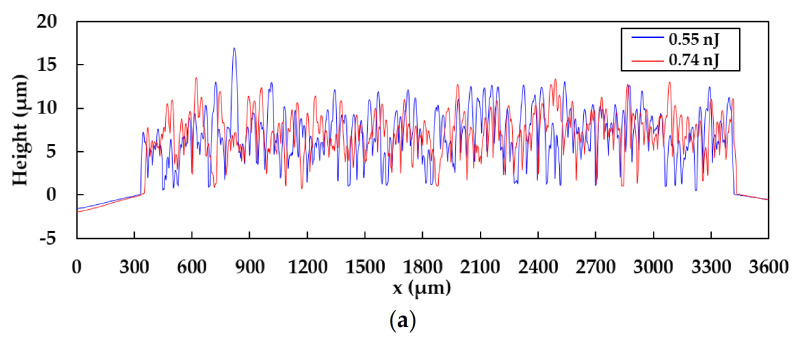

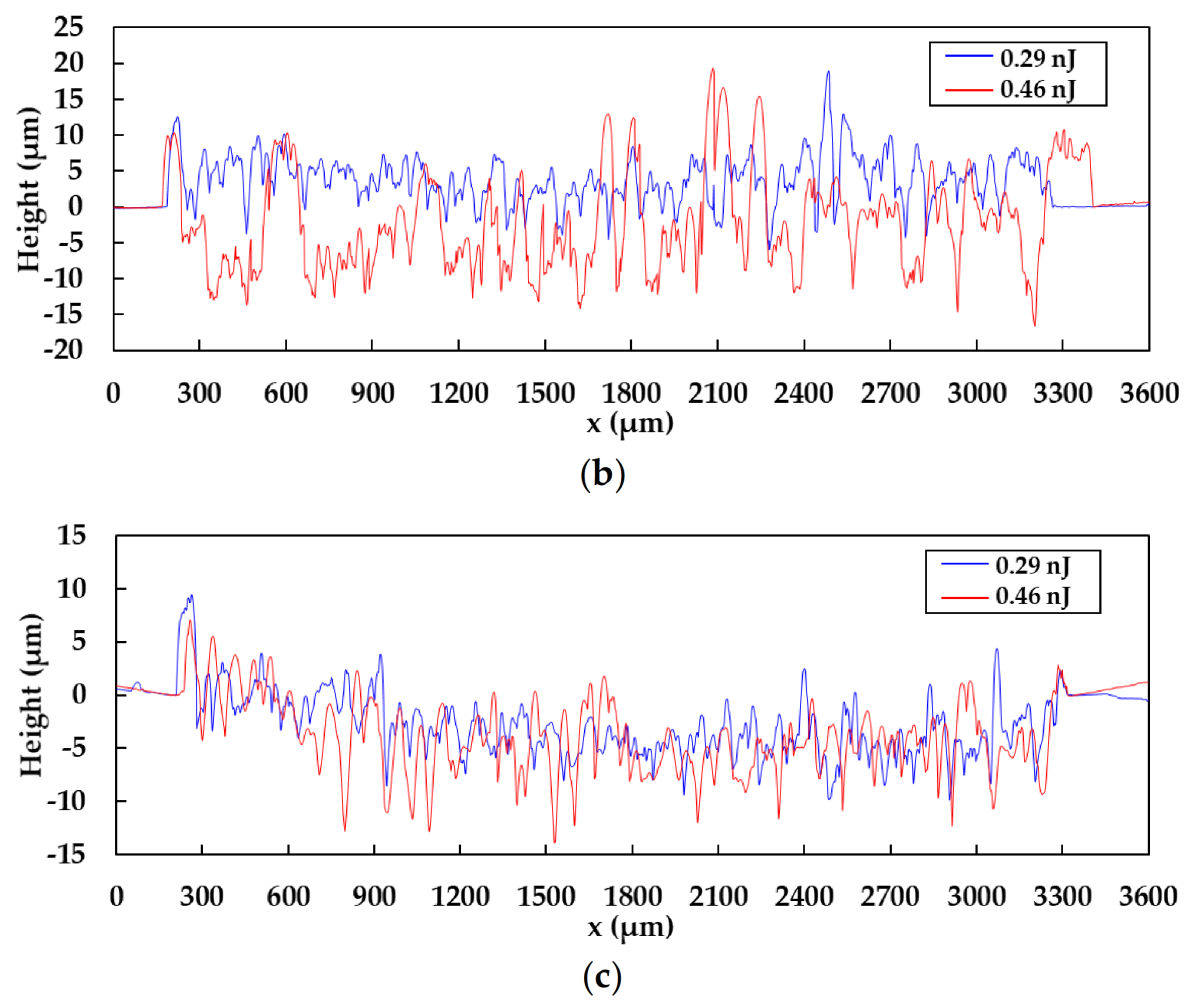
Cross-sectional profiles of the patterns fabricated at a pulse energy of (**a**) 0.55 and 0.74 nJ on glass substrates, and at a pulse energy of 0.29 and 0.46 nJ on (**b**) PEN and (**c**) PET substrates.

**Figure 9:**
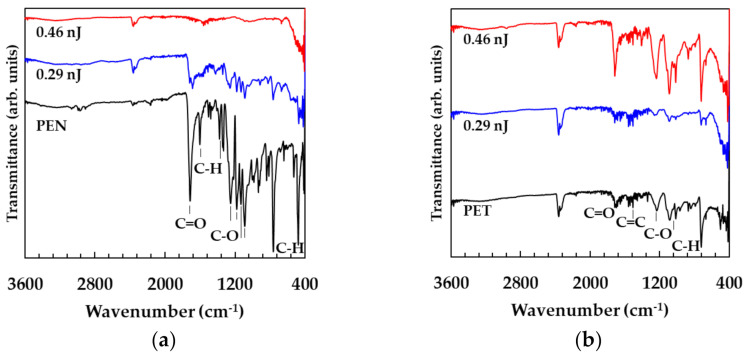
FT-IR spectra of the patterns on (**a**) PEN and (**b**) PET substrates.

To evaluate the damage of the PEN and PET substrates, the cross-sectional profiles of the patterns (5 × 3 mm^2^) were measured as shown in Figure 8a–c. The cross-sectional profiles were vertical to the raster scanning direction. The cross-sectional profiles of the patterns on glass substrates were not dependent on the pulse energy, as shown in Figure 8a. However, the cross-sectional profiles of the patterns fabricated at a pulse energy of 0.46 nJ on PEN and all the patterns on PET substrates exhibited damages on the substrates because the heights of the patterns were lower than the surface levels of the substrates. These results suggest that the thermal volatilization was caused by irradiating excess pulse energy. The intense adhesion of the patterns on PEN and PET substrates also agreed with the results that the patterns and the substrates reacted chemically. In addition, the ATR-FTIR spectra of the patterns on PEN and PET substrates are shown in Figure 9a,b to evaluate the damages of the polymer substrates. The absorption peaks corresponding to the PEN decreased in the case of the pulse energy of 0.46 nJ. The absorption peaks corresponding to the PET increased at the high pulse energy of 0.46 nJ compared to the low pulse energy of 0.29 nJ. This result was consistent with the previous report that the absorption of PET improved by heating at high temperatures [29].

In comparison with the Co-composite patterns fabricated on the glass substrates, the patterns fabricated on the PET and PEN substrates were more easily oxidized, but metal Co remained in the patterns. Such spatially selective sintering process of a Co composite is an effective method to generate the Co-based catalysts in various gas and glucose sensors. In addition, the Co-based micropatterns will be used not only for direct application as microelectronics, but also as blanks for further modification, as shown earlier [30]. Here, femtosecond laser pulses were used for reductive sintering of Co_3_O_4_ nanoparticles because the total irradiated energy is expected to be reduced for the sintering using the high intensity of the laser pulses. The pulse duration of the femtosecond laser pulses is shorter than the duration of the thermal diffusion, resulting that the total irradiated energy can be reduced for the sintering of the nanoparticles. By considering the thermal diffusion, a picosecond pulsed laser is also a candidate for the reductive sintering.

## 4. Conclusions

Cobalt/cobalt oxide composite patterns were formed on glass, PEN, and PET substrates via the femtosecond laser reductive sintering of cobalt oxide nanoparticles in an ambient atmosphere.

(1) A Co_3_O_4_ nanoparticle ink, composed of the Co_3_O_4_ nanoparticles, ethylene glycol as a reductant, and PVP as a dispersant, was spin-coated on the substrates. The ink film exhibited sufficient absorption at the wavelength of the near-infrared femtosecond laser pulses.

(2) Co/CoO composites were fabricated on glass substrates without significant generation of Co_3_O_4_. In contrast, Co/CoO/Co_3_O_4_ composites were formed on both the PEN and PET substrates. These results suggest that the polymer substrates with low thermal resistance reacted with the patterns and oxidized the patterns to a greater extent than occurred with the patterns on glass substrates.

Such direct writing technique of the cobalt/cobalt oxide composites is useful for spatially selective printing of the catalysts and detectors of the functional microsensors.

## Figures and Tables

**Table 1:** Physical parameters of the substrates.

	Thickness (mm)	Thermal Conductivity (W/K·m)	Heat Capacity (J/K·kg)	Density (kg/m^3^)	Glass Transition Temperature (°C)	Melting Point (°C)	Ref.
Glass	1	1.38	703	2203	~1700	>1700	[21]
PEN (Teonex, Teijin)	~0.25	~0.2	0.87	~1330	121	269	[22]
PET (Lumirror, Toray)	~0.25	~0.14	0.32	~1400	155	263	[23]

## Data Availability

Not applicable.
